# Audit on Management of Behavioural and Psychological Symptoms Among Dementia Patients in Humber Older People's Mental Health Services

**DOI:** 10.1192/bjo.2024.523

**Published:** 2024-08-01

**Authors:** Sunday Adeoye

**Affiliations:** Humber NHS Trust, Hull, United Kingdom

## Abstract

**Aims:**

Up to 75% of dementia patients will experience behavioural (non-cognitive) symptoms in their lifetime. Therefore, it is important to ensure delivery of high level of quality care to these set of patients.

The NICE guideline recommends that:

1. Non-pharmacological method should be used before pharmacological method in the management of behavioural symptoms.
2. When antipsychotics are used, they should be started at low dose and increased slowly.
3. Those started on antipsychotics should have follow up at least 6 weeks after commencement.

Aim: The audit aims to compare the care we give dementia patients with behavioural symptoms against the NICE guideline.

The objectives are:

1. To assess use of non-pharmacological method before pharmacological method in the management of behavioural symptom in dementia patients.
2. To assess antipsychotic prescriptions in the management of behavioural symptoms in dementia patients.
3. To assess if patients started on antipsychotics were properly followed up.

**Methods:**

Electronic records of 34 patients who met the inclusion criteria were assessed and information related to the objectives were extracted. Data was stored securely in the trust laptop. Analysis of the information was done using Microsoft Excel version 2022. Results were presented in charts.

**Results:**

The result showed that the commonest behavioural symptoms reported was agitation and verbal aggression which accounted for 34% and 29% respectively. About 24% of the patient were commenced on medication for their symptoms without trial of non-pharmacological methods. Out of the patient that were on medications, risperidone was the commonest medication prescribed accounting for 37%. Other medications prescribed included quetiapine, amisulpride and lorazepam. The result also showed that those started on medication were properly followed up according to the NICE guideline.

**Conclusion:**

The audit showed that the NICE guideline is not fully followed, adherence to the guideline is around 75% overall. Efforts should be geared toward enlightening professionals about the need to follow the NICE guideline in managing this condition. It would be worthwhile to re-audit in 12 to 24 months.

